# An unusual cause of small bowel obstruction: duodenal bezoar-induced diverticulitis

**DOI:** 10.1093/jscr/rjaf853

**Published:** 2025-11-03

**Authors:** Sophia Chan

**Affiliations:** Department of General Surgery, Logan Hospital, Armstrong Rd & Loganlea Rd, Meadowbrook, QLD 4131, Australia

**Keywords:** enterolithiasis, bowel obstruction, diverticulitis, perforation

## Abstract

Small bowel obstruction (SBO) secondary to enterolithiasis in the setting of small bowel diverticulosis is rare, and diagnosis can be challenging. Enteroliths may cause complications including diverticulitis, perforation, and obstruction, typically requiring surgical removal. An 83-year-old woman presented with abdominal pain, vomiting, and constipation. CT imaging demonstrated an intraluminal lesion at the duodenojejunal junction adjacent to an inflamed duodenal diverticulum, consistent with diverticulitis and partial SBO. Initial conservative management included nasogastric decompression, intravenous antibiotics, and parenteral nutrition. Serial imaging showed migration of the intraluminal mass to the proximal jejunum, but persistent obstruction prompted laparotomy and enterotomy, revealing an inspissated food bezoar. Enterolith-induced SBO and diverticulitis is an uncommon but important differential in elderly patients presenting with obstructive symptoms. While conservative management may be attempted in select patients, progression to surgery is frequently required. Early recognition and tailored management can improve outcome.

## Introduction

Small bowel obstruction (SBO) is a common surgical pathological process. The most prevalent causes include adhesions, hernias, malignancy, inflammatory bowel disease, foreign bodies, and volvulus [1]. Rarely, obstruction is due to an enterolith formed within a small bowel diverticulum [2].

Small bowel diverticulosis is far less prevalent than colonic diverticulosis and is often detected incidentally, with incidence increasing with age [3]. Diverticula (sac-like protrusions) are thought to arise from mucosal herniation through areas of muscular weakness under high intraluminal pressure [2]. Complications may include including diverticulitis, bleeding, perforation, biliary or pancreatic disease, intestinal obstruction, abscess formation, malabsorption, anaemia, or volvulus [3].

Enterolithiasis refers to the formation of gastrointestinal concretions and can be classified as primary (intraluminal, often within sites of stasis such as diverticula, blind pouches, or strictures) or secondary (migration and/or fistulization of extraluminal stones, e.g. gallstones, renal calculi). Large or obstructive enteroliths can precipitate diverticulitis, perforation, haemorrhage, intussusception, or bowel obstructions [4].

Management typically involves enterolith removal and treatment of the underlying precipitating factor as a preventative measure. In cases of a bowel obstruction, decompression with a nasogastric tube, resuscitation with intravenous fluids and electrolyte replacement is vital. Surgical intervention remains the most definitive approach—either by fragmentation and milking of the pieces into the large bowel or proximal enterotomy of the non-oedematous segment and manual removal of enterolith. Alternatively, endoscopic segment dilatation and stone retrieval or endoscopic electrohydraulic or mechanical lithotripsy can be considered [4].

We present the case of an 83-year-old woman with a duodenal enterolith complicated by duodenal diverticulitis and partial SBO, ultimately requiring surgical intervention.

## Case presentation

An 83-year-old woman presented with a 4-day history of intermittent, crampy abdominal pain, and 2 days of non-bilious vomiting. She reported 1 week of constipation, with her last bowel motion 3 days prior. Past surgical history included laparoscopic cholecystectomy (10 years prior) and total abdominal hysterectomy with bilateral salpingo-oophorectomy (50 years prior). Medical history included hypertension, hyperthyroidism, and glaucoma. She was otherwise independent, still driving and lived alone in the community.

**Figure 1:**
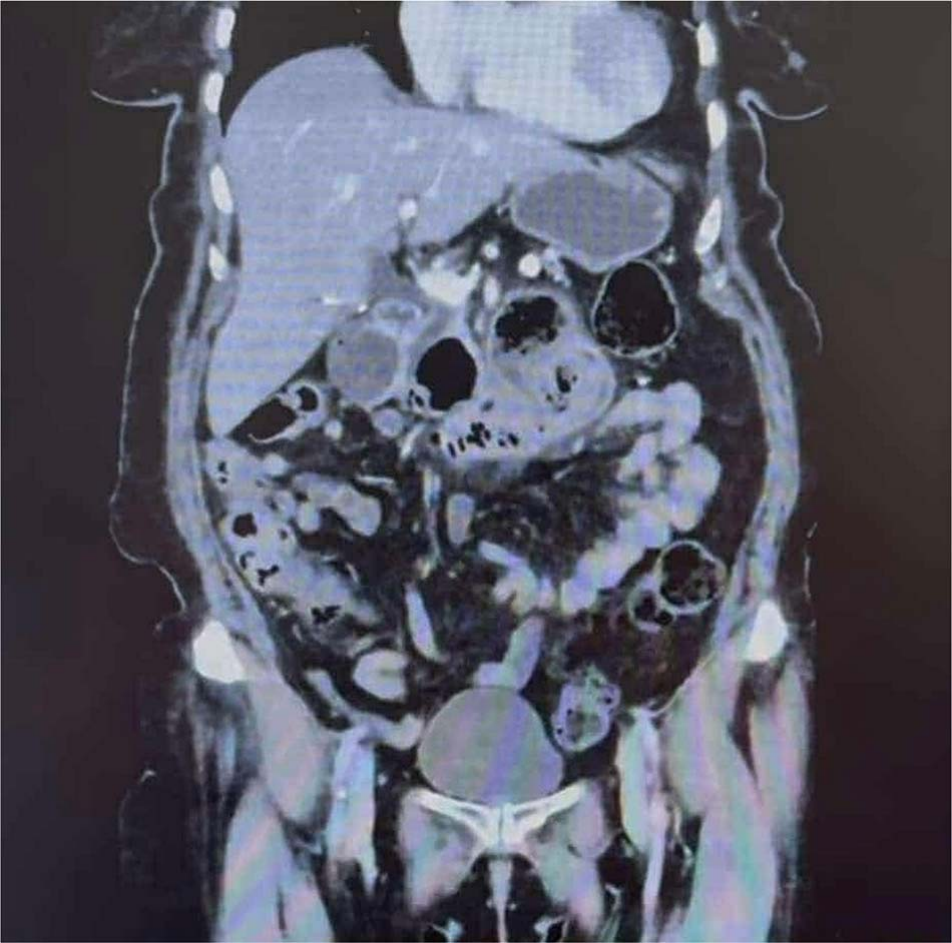
CT abdomen and pelvis demonstrating a partially obstructing intraluminal lesion at the duodeno-jejunal junction, adjacent to an inflamed duodenal diverticulum raising suspicion for an impacted food bolus, a mass lesion or intussusception. There was no evidence of perforation, free gas, or fluid.

On arrival to the Emergency Department, the patient was hypertensive, however, all other vitals were within normal limits. On examination, the patient was dehydrated and focally tender in the epigastrium without peritonism. Laboratory results showed a mildly elevated white cell count of 11.4 × 10^9^/L and a normal lactate of 1.7. Computer tomography (CT) imaging (Fig. 1) demonstrated a partially obstructing intraluminal lesion at the duodeno-jejunal junction, adjacent to an inflamed duodenal diverticulum raising suspicion for an impacted food bolus, a mass lesion or intussusception. There was no evidence of perforation, free gas or fluid.

Given the patient was clinically well, she was kept nil by mouth and managed conservatively with a nasogastric tube, intravenous pantoprazole and fluids. The imaging was discussed with a surgical colleague for a second opinion and the impression was consistent with acute duodenal diverticulitis likely secondary to a food bolus impacted in the involved diverticulum. Subsequently, intravenous piperacillin-tazobactam was commenced for broad-spectrum antibiotic coverage. A Gastrografin contrast study showed passage of oral contrast beyond the food bolus, and the patient passed flatus, prompting cautious dietary progression.

Unfortunately, the patient experienced intractable nausea, intolerance to oral intake and significantly high nasogastric tube outputs. Parenteral nutrition was commenced to prevent malnutrition. A repeat CT on day nine showed migration of the intraluminal mass to the proximal jejunum with new proximal dilation, suggesting partial SBO. A further scan 2 days later confirmed persistent obstruction with no further migration.

**Figure 2:**
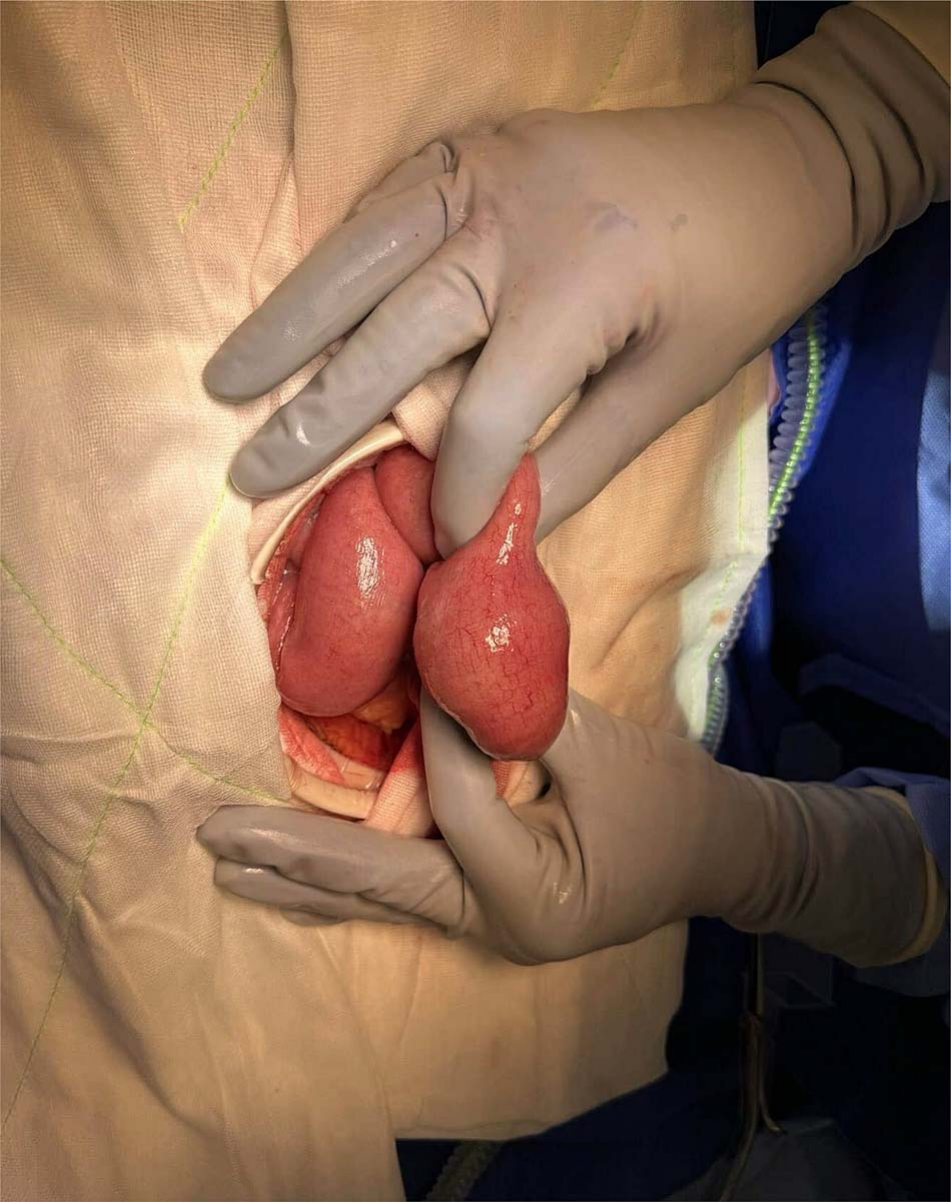
Intraoperative image of segment of small bowel containing enterolith.

**Figure 3:**
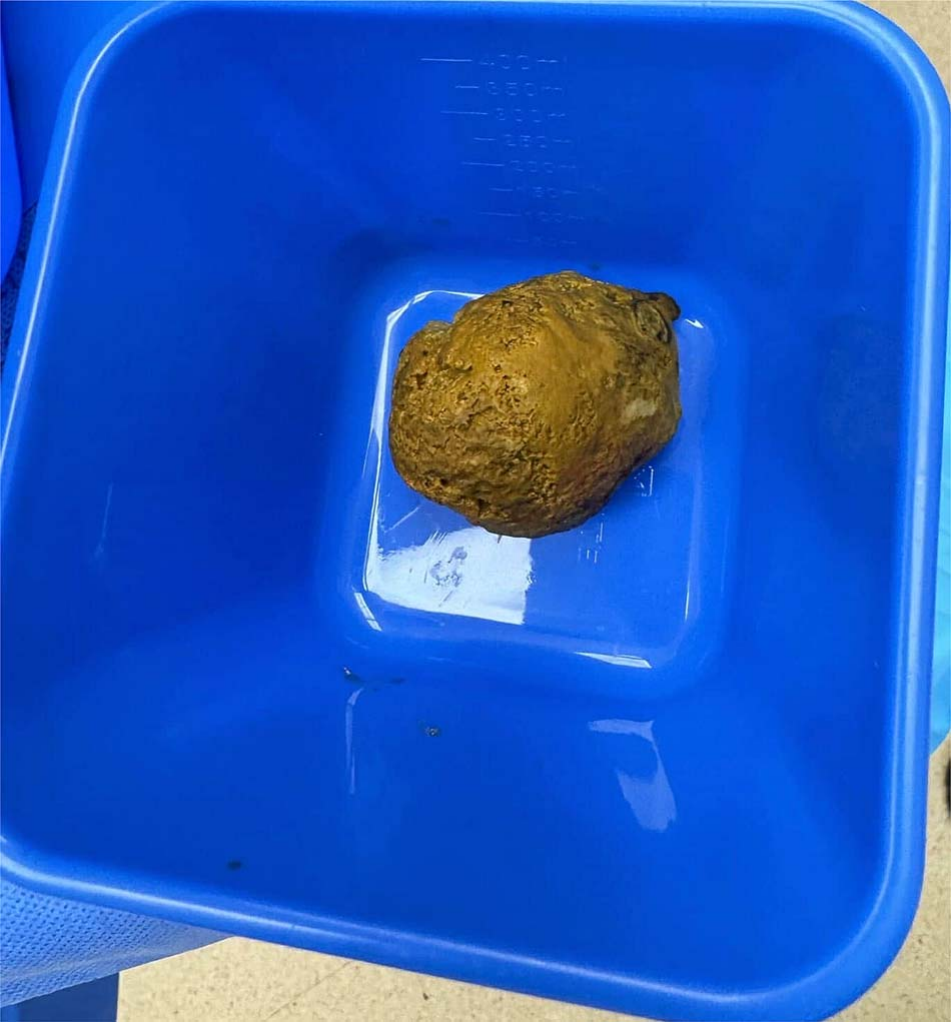
Inspissated material (enterolith) milked from the small bowel after enterotomy.

Given worsening obstruction, a laparotomy and small bowel enterotomy with extraction was performed (Fig. 2). Intraoperative findings revealed an inspissated mass of partially digested food in the proximal–mid jejunum (Fig. 3). The postoperative course was complicated by intractable nausea, peripheral oedema, malignant hypertension, and a PICC-associated 6 cm basilic vein thrombus, but bowel function returned with passage of stool and flatus. Erythromycin was initiated as a prokinetic.

The patient underwent a laparotomy and enterotomy for removal of food bezoar. Intra-operative findings were consistent with an inspissated mass of partially digested food in the proximal to mid-jejunum. The postoperative recovery was prolonged and complicated by intractable nausea, peripheral oedema, and malignant hypertension. Reassuringly, the patient had opened her bowels several times and passed flatus. She was discharged on day 27 and remained well at two-month follow-up.

## Discussion

Small bowel diverticulosis is rare, and complications such as diverticulitis or SBO secondary to enterolithiasis are even more uncommon.

A review of the existing literature identified only nine documented cases of concurrent small bowel diverticulitis and bowel obstruction attributable to enterolithiasis. The mean age of presentation was 81 years, with a male to female predominance (2:1). All cases were managed surgically, with a 77% frequency for laparotomy and the remaining 23% via laparoscopic-assisted approach. The vast majority of cases involved an enterotomy to retrieve the enterolith, followed by a bowel resection and anastomosis. Preoperative diagnosis was challenging in most cases, prompting emergency surgery. Only one reported case involved initial conservative management, which ultimately failed, necessitating delayed surgery.

Our patient’s management deviated from most reported cases due to her age, comorbidities and partial nature of the obstruction. An initial trial of conservative management was supported by CT evidence of enterolith migration and absence of complete obstruction. However, symptom persistence and progression ultimately necessitated operative intervention.

This case reinforces the importance of tailoring management to patient-specific risk profiles and clinical progression, supported by serial imaging. It also highlights that while conservative management may be appropriate in select cases, progression to surgery is often required.

## Conclusion

Enterolith-induced small bowel diverticulitis and obstruction is a rare but important diagnostic consideration in elderly patients presenting with a SBO, particularly in those with risk factors for small bowel stasis. Preoperative diagnosis is challenging, and management strategies must balance the risks of surgery against the potential for resolution with conservative measures. Increased clinician awareness may facilitate earlier diagnosis and appropriate intervention, potentially improving outcomes in this uncommon but significant pathology.
